# Supplementary material for: Discovery of NSD2 non-histone substrates and design of a super-substrate
Source: Commun Biol. 2024 Jun 8;7:707. doi: 10.1038/s42003-024-06395-z (PMC11162472; doi:10.1038/s42003-024-06395-z)
Supplement: Supplementary file 2 — Supplementary Information [file 42003_2024_6395_MOESM2_ESM.pdf]

Discovery of new NSD2 non-histone substrates and design of a super-substrate

Sara Weirich, Denis Kusevic, Philipp Schnee, Jessica Reiter, Jürgen Pleiss & Albert Jeltsch*

Supplementary Information

Supplementary Figures

Supplementary Figure 1. Investigation of the protein quality and methylation activity of NSD2.

Supplementary Figure 2. Additional information related to Figure 1.

Supplementary Figure 3. Additional data related to Figure 2c.

Supplementary Figure 4. Comparison of the methylation level at peptides when P30 and P43 were replaced by G and S.

Supplementary Figure 5. Comparison of the methylation of H3K36-GST and ssK36-GST by NSD2.

Supplementary Figure 6. NSD2 specifically methylates the NSD2 super-substrate but not a previously designed super-substrate for SETD2.

Supplementary Figure 7. Additional information related to the MD simulations of NSD2.

Supplementary Figure 8. Purification, Western Blot analysis and methylation of NSD2 non-histone substrate candidate proteins.

Supplementary Figure 9. Additional protein methylation data with ATRX and FANCM allowing to compare their methylation with H3.

Supplementary Figure 10. Validation of the anti-H3K36me1 antibody for detection of methylated ATRX and FANCM proteins.

Supplementary Figure 11. Immunoblot detection of the expression of NSD2 full-length, ATRX and FANCM in HEK293 cells.

Supplementary Figure 12: Uncropped images of the Figures and Supplementary Figures.

Supplementary Tables

Supplementary Table 1: Sequences of the peptide SPOT array shown in Figure 2a.

Supplementary Table 2: Sequences of the peptide SPOT array shown in Figure 2b.

Supplementary Table 3: Sequences of the peptide SPOT array shown in Figure 2c.

Supplementary Table 4: Sequences of the peptide SPOT array shown in Figure 6b.

Supplementary Table 5: Compilation of the domain boundaries and properties of the cloned protein methylation substrate candidates

Supplementary Table 6: Additional information about the MD simulated systems.

Supplementary References

Supplementary Figures

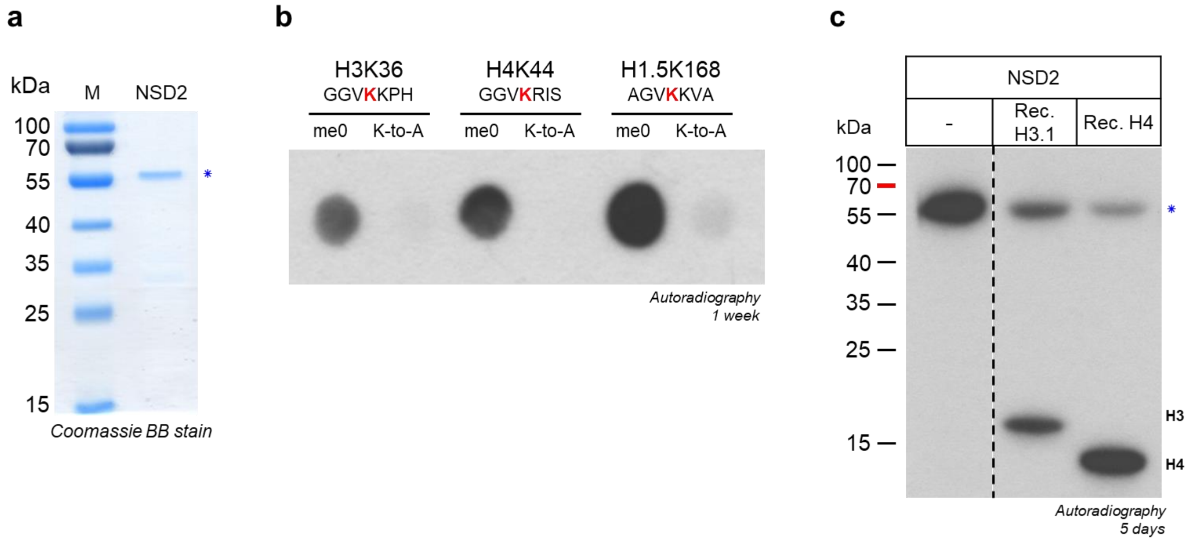

Supplementary Figure 1. Investigation of the protein quality and methylation activity of NSD2. a Coomassie stained gel of the purified GST tagged NSD2 (aa 991-1240). **b** Peptide array containing 15 aa long peptides of H3K36 (29-43, APATGGVKKPHRYRP), H4K44 (37-51, LARRGGVKRISGLIY) and H1.5K168 (161-175, KPAAAGVKKVAKSPK) and the corresponding K-to-A mutations as negative controls were incubated with NSD2 in the presence of radioactively labeled AdoMet as cofactor. The autoradiographic image after exposure for one week confirmed the methylation of H3K36, H4K44, H1.5K168 and loss of methylation for the negative controls. **c** Recombinant H3.1 (1.6 µM) and H4 (2.2 µM) were methylated by NSD2 (2.5 µM) using radioactively labeled AdoMet. To investigate potential automethylation of NSD2, the same experiment was performed without substrate. Methylated samples were separated by SDS-PAGE and analyzed by autoradiography after 5 days of film exposure. The corresponding bands of the methylated H3 and H4 are marked in red. Automethylation of NSD2 is labelled with a blue asterisk. Both parts were taken from the same gel image.

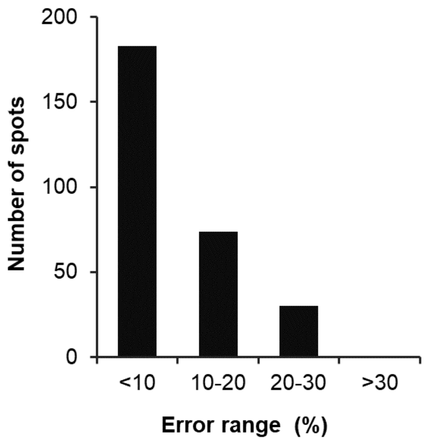

Supplementary Figure 2. Additional information related to Figure 1. The distribution of the standard errors of the mean of NSD2 activity is given for all peptides tested in the three independent substrate specificity arrays used for the averaged data shown in Figure 1a and b.

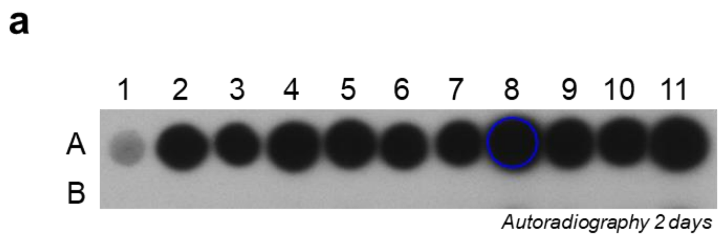

Spot position	Sequence
A1 [H3K36]	A P A T G G V K K P H R Y R P
A8	A P K T G G V K R P N N Y R P

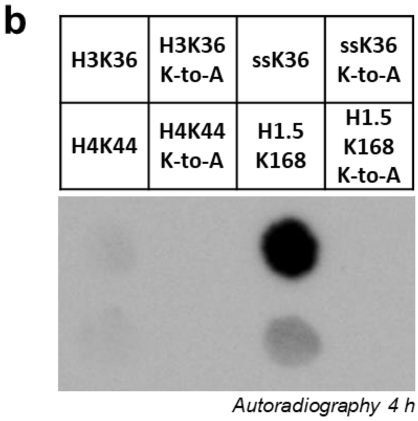

Supplementary Figure 3. Additional data related to Figure 2C and comparison of ssK36 with other substrates. a longer film exposure of the NSD2 methylated peptide SPOT array shown in Figure 2c reveals a weak methylation signal at the H3K36 peptide. **b** Peptide array methylation experiment similar as in Supplementary Figure 1b, showing that the NSD2 super-substrate (ssK36, AGKTGGVKRPNNYRS) peptide is methylated much better than H3K36, H4K44 and H1.5K168 peptides.

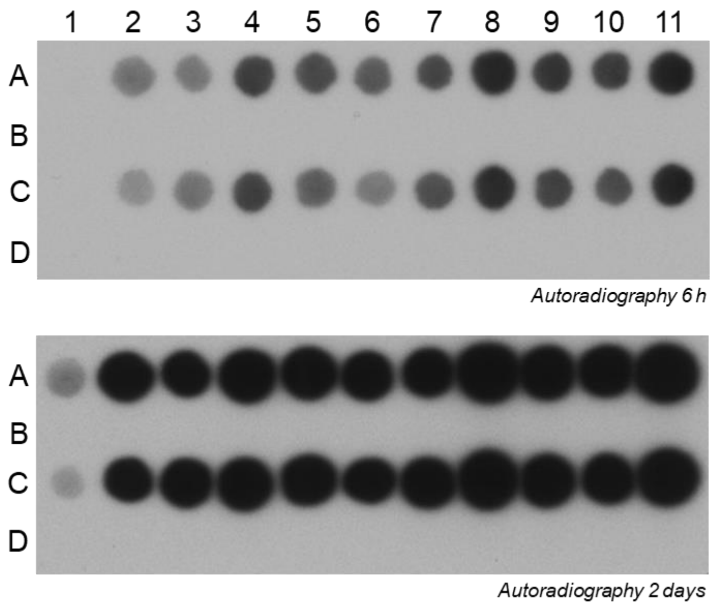

Spot	ID	Sequence
A1	H3K36	A P A T G G V K K P H R Y R P
A2	2 mutations	A P A T G G V K K P N N Y R P
A3	3 mutations	A P K T G G V K R P H N Y R P
A4		A P K T G G V K K P N N Y R P
A5		A P A T G G V K R P N N Y R P
A6		A P A T G G V K K V N N Y R P
A7	4 mutations	A P K T G G V K R V H N Y R P
A8		A P K T G G V K R P N N Y R P
A9		A P K T G G V K K V N N Y R P
A10		A P A T G G V K R V N N Y R P
A11	5 mutations	A P K T G G V K R V N N Y R P
B1	H3K36A	A P A T G G V A K P H R Y R P
B2	2 mutations_K36A	A P A T G G V A K P N N Y R P
B3	3 mutations_K36A	A P K T G G V A R P H N Y R P
B4		A P K T G G V A K P N N Y R P
B5		A P A T G G V A R P N N Y R P
B6		A P A T G G V A K V N N Y R P
B7	4 mutations_K36A	A P K T G G V A R V H N Y R P
B8		A P K T G G V A R P N N Y R P
B9		A P K T G G V A K V N N Y R P
B10		A P A T G G V A R V N N Y R P
B11	5 mutations_K36A	A P K T G G V A R V N N Y R P

Spot	ID	Sequence
C1	H3K36_GS	A G A T G G V K K P H R Y R S
C2	2 mutations_GS	A G A T G G V K K P N N Y R S
C3	3 mutations_GS	A G K T G G V K R P H N Y R S
C4		A G K T G G V K K P N N Y R S
C5		A G A T G G V K R P N N Y R S
C6		A G A T G G V K K V N N Y R S
C7	4 mutations_GS	A G K T G G V K R V H N Y R S
C8		A G K T G G V K R P N N Y R S
C9		A G K T G G V K K V N N Y R S
C10		A G A T G G V K R V N N Y R S
C11	5 mutations_GS	A G K T G G V K R V N N Y R S
D1	H3K36A_GS	A G A T G G V A K P H R Y R S
D2	2 mutations_GS_K36A	A G A T G G V A K P N N Y R S
D3	3 mutations_GS_K36A	A G K T G G V A R P H N Y R S
D4		A G K T G G V A K P N N Y R S
D5		A G A T G G V A R P N N Y R S
D6		A G A T G G V A K V N N Y R S
D7	4 mutations_GS_K36A	A G K T G G V A R V H N Y R S
D8		A G K T G G V A R P N N Y R S
D9		A G K T G G V A K V N N Y R S
D10		A G A T G G V A R V N N Y R S
D11	5 mutations_GS_K36A	A G K T G G V A R V N N Y R S

Supplementary Figure 4. Comparison of the methylation levels of peptides when P30 and P43 were replaced by G and S. As the H3 peptide contains P at position 2 and at the end, we were concerned that this may cause artefacts. Therefore, in some of the design steps, P30 and P43 were replaced by G and S. This array shows a direct comparison of the methylation of PP and GS peptides in the design step shown in Figure 2c, indicating that the PP/GS change does not affect the peptide methylation.

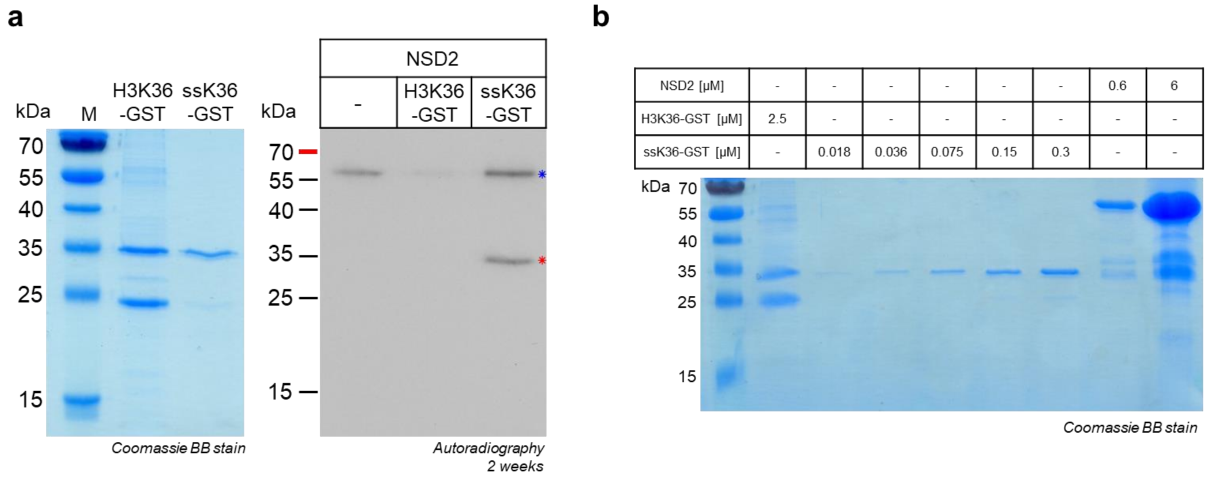

Supplementary Figure 5. Comparison of the methylation of H3K36-GST and ssK36-GST by NSD2. Direct comparison of the natural H3 sequence and the NSD2 specific H3K36 super-substrate (ssK36) reveals 4 amino acid changes: A31K, K37R, H38N and R39N. **a** GST-tagged H3K36 (29-43) and ssK36 (29-43) were overexpressed and purified. For the protein methylation assay, 2.5 µM H3K36-GST or 0.3 µM ssK36-GST, were incubated with 0.5 µM NSD2 in the presence of radioactively labelled AdoMet and separated by SDS-PAGE. Methylation was analyzed by autoradiography after 2 weeks of film exposure. The methylated ssK36-GST band is marked with a red asterisk. Automethylation of NSD2 is labelled with a blue asterisk. The gels used for Coomassie staining and autoradiography were loaded and run in parallel using the same protein amounts. **b** Protein loading gel run in parallel to the methylation experiment in Figure 2e using the same protein amounts.

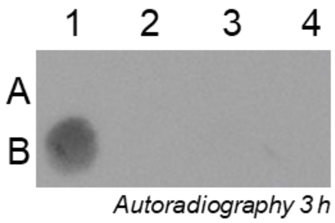

Spot position	ID	Sequence
A1	H3K36	A P A T G G V K K P H R Y R P
A2	H3K36A	A P A T G G V A K P H R Y R P
A3	H3K36_GS	A G A T G G V K K P H R Y R S
A4	H3K36A_GS	A G A T G G V A K P H R Y R S
B1	ssK36 (NSD2)	A G K T G G V K R P N N Y R S
B2	ssK36A (NSD2)	A G K T G G V A R P N N Y R S
B3	ssK36 (SETD2)	A P R F G G V K R P N R Y R P
B4	ssK36A (SETD2)	A P R F G G V A R P N R Y R P

Supplementary Figure 6. NSD2 specifically methylates the NSD2 super-substrate but not a previously designed super-substrate for SETD2. The NSD2 super-substrate is designated ssK36 (NSD2) in this table, the previously designed and investigated super-substrate for SETD2 [1,2] is designated ssK36 (SETD2). The exchange of P30 and P43 by G and S in the ssK36 (NSD2) has no effect on enzyme activity as shown in Supplementary Figure 4.

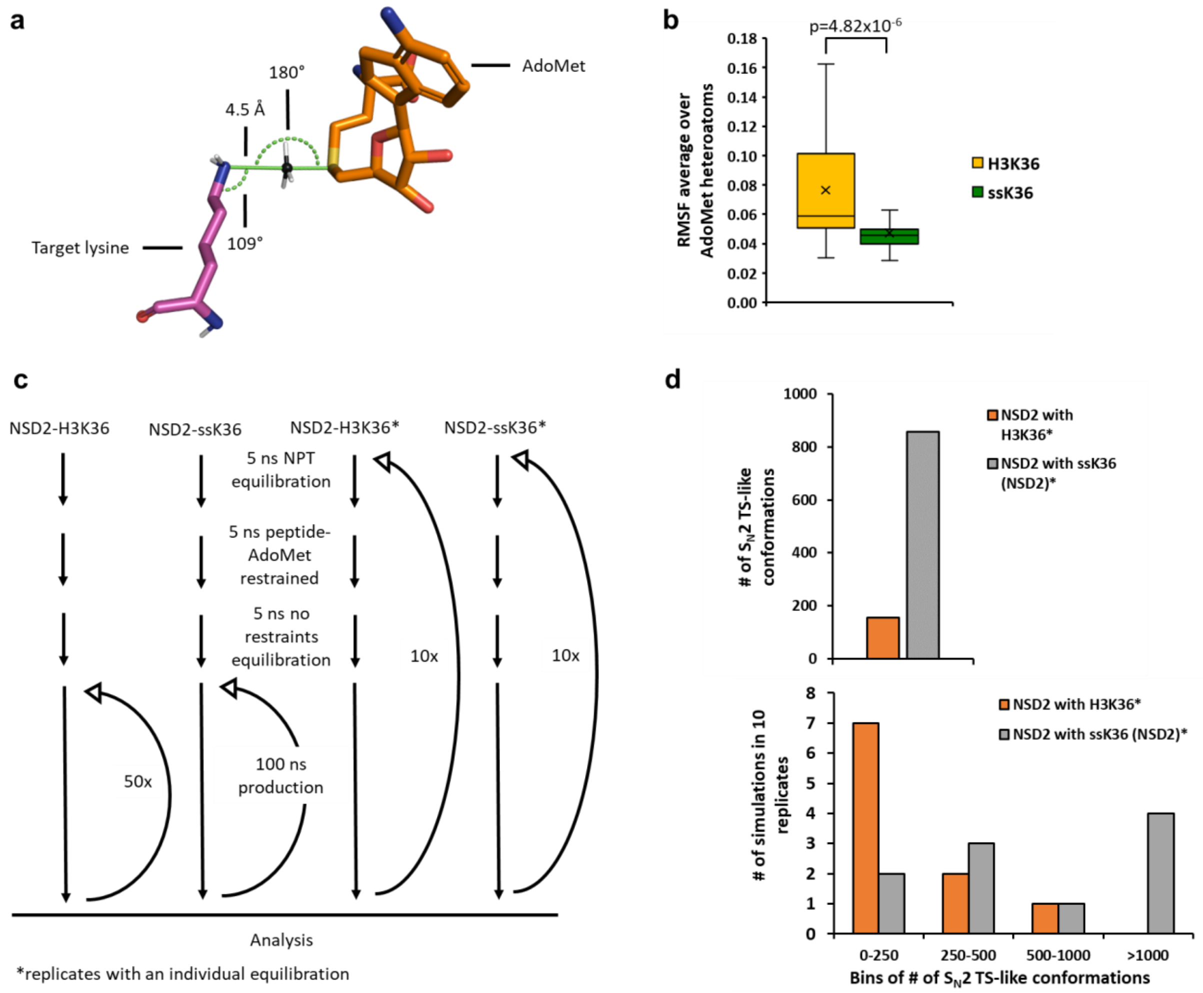

Supplementary Figure 7. Additional information related to the MD simulations of NSD2. a Criteria used for definition of a successful docking event derived from the geometry of the S_N2 TS-like conformation of PKMTs [3]. **b** Flexibility of AdoMet bound in NSD2-H3K36-AdoMet or NSD2-ssK36-AdoMet complexes. The flexibility was described as the root square of the mean AdoMet heteroatom fluctuation (RMSF) observed in the 50 independent simulations of both complexes. Boxes show the median, 1st and 3rd quartile. Whiskers display the 1.5 IQR distance (n=50 independent MD simulations). The p-value was determined by a two-sided T-test with unequal variance. **c** Schematic representation of the different MD simulation settings. **d** Analysis of the MD simulation replicates in which each of the 10 replicates had an individual equilibration phase (labeled by * in panel c).

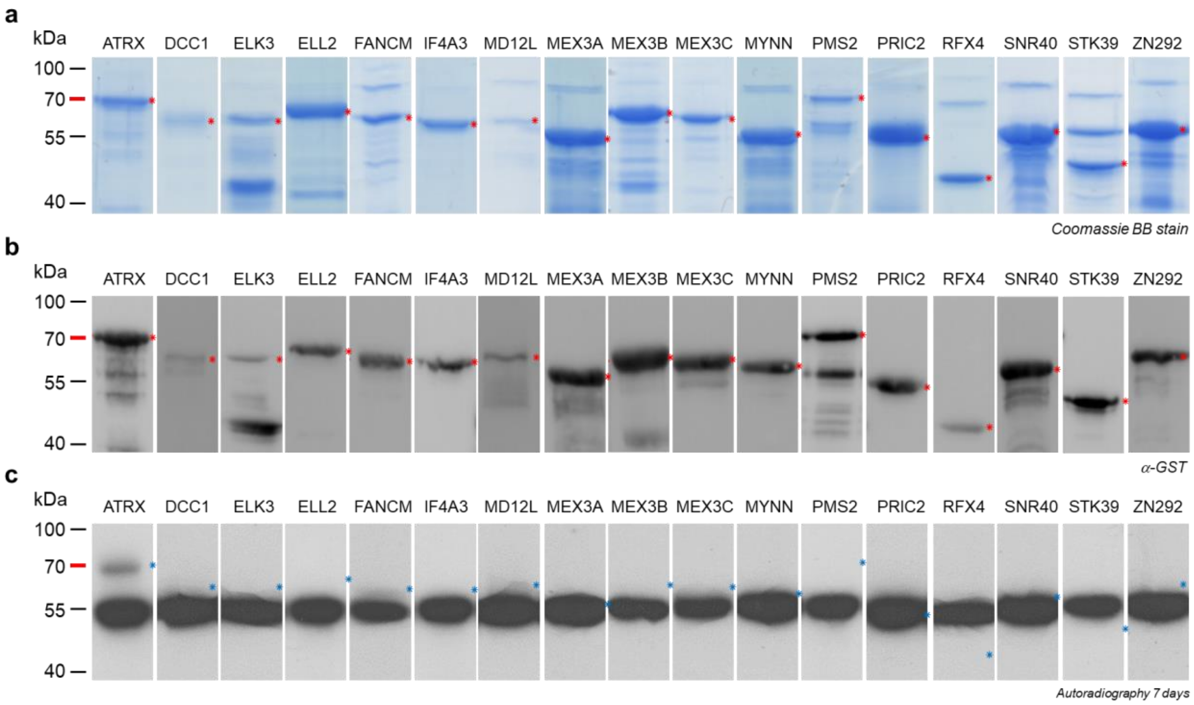

Supplementary Figure 8. Purification, Western Blot analysis and methylation of NSD2 non-histone substrate candidate proteins. a Coomassie stained SDS gel of 17 potential NSD2 substrate proteins. **b** Western Blot analysis of the purified proteins using an anti-GST antibody. The protein bands of expected size are labelled with red asterisks. **c** Purified proteins were methylated by NSD2. The autoradiographic image after 7 days of exposure shows strong automethylation signals of NSD2 and blue asterisks indicate expected methylation signals of the different non-histone proteins. Information about the domain boundaries and properties of the cloned target proteins is provided in Supplementary Table 5.

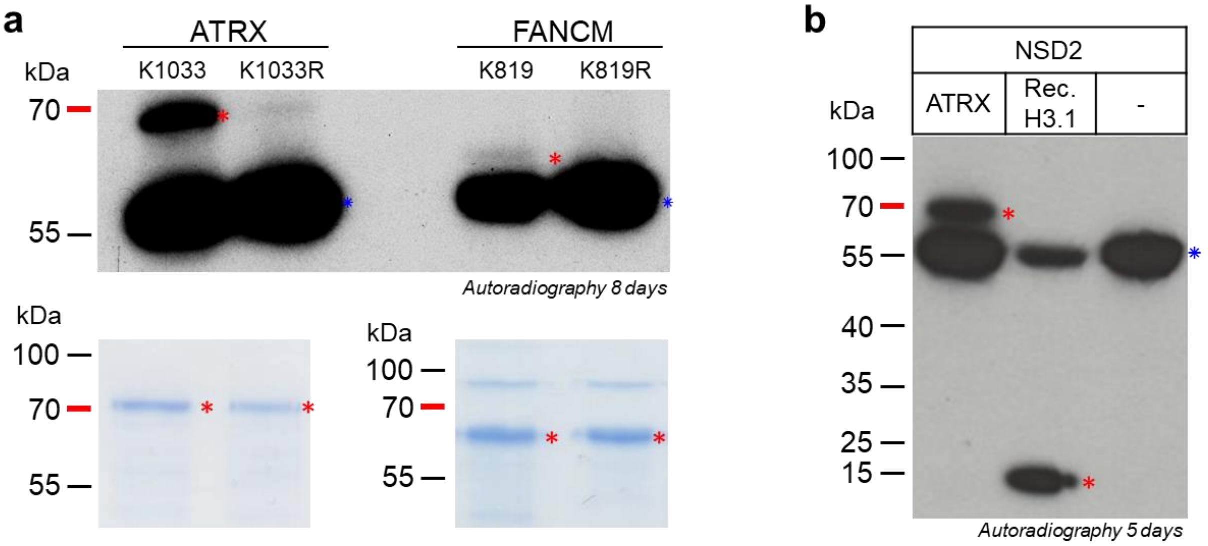

Supplementary Figure 9. Additional protein methylation data with ATRX and FANCM allowing to compare their methylation with H3. a The methylation reactions shown in Figure 6c were taken from the same gel, directly allowing to compare the intensity of the methylation of ARTX and FANCM. NSD2 concentration was 2.5 µM, ATRX and FANCM concentrations were 6 and 3 µM. The ATRX and FANCM bands are marked with red asterisks. Automethylation of NSD2 is labelled with a blue asterisk. **b** Comparison of the methylation of ATRX and recombinant H3. NSD2 concentration was 2.5 µM, ATRX and recombinant H3.1 concentrations were 3 and 1.6 µM. The methylated ATRX and H3.1 bands are marked with red asterisks. Automethylation of NSD2 is labelled with a blue asterisk.

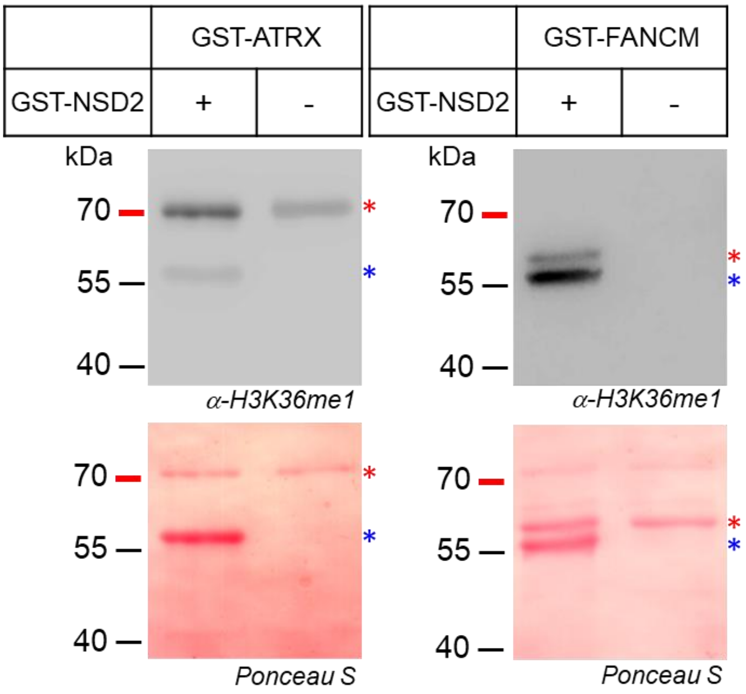

Supplementary Figure 10. Validation of the anti-H3K36me1 antibody for detection of methylated ATRX and FANCM proteins. ATRX and FANCM proteins were incubated with or without NSD2 in the presence of unlabeled AdoMet as methyl group donor, followed by SDS-PAGE and Western Blot analysis. Ponceau S staining of the methylated and unmethylated protein substrates is shown in the lower panel. Western blot of the transferred methylated and unmethylated protein substrates probed with the anti-H3K36me1 antibody is shown in the upper panel. The corresponding bands of the expected size are marked with a red asterisk. Automethylation of NSD2 is labelled with a blue asterisk.

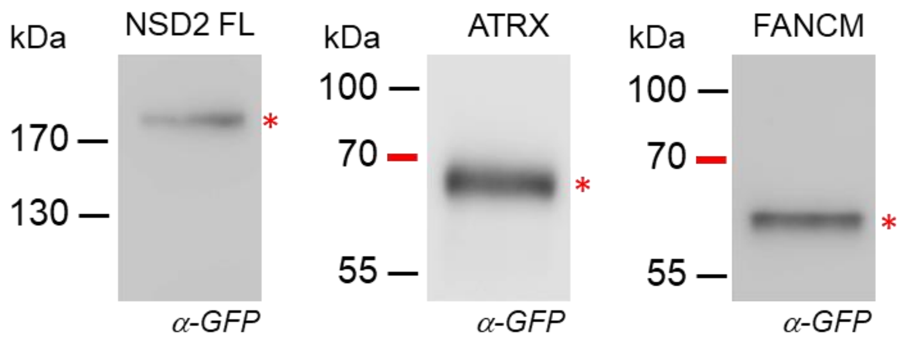

Supplementary Figure 11. Immunoblot detection of the expression of NSD2 full-length, ATRX and FANCM in HEK293 cells. The expressed proteins were detected by probing with an anti-GFP antibody. The corresponding bands of the expected size are marked with a red asterisk.

Supplementary Figure 12: Uncropped images of the Figures and Supplementary Figures.

- Related to Figure 2d

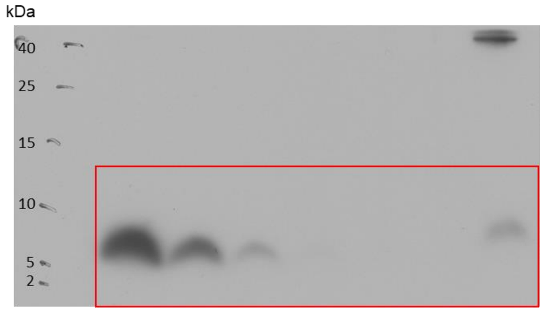

- Related to Figure 2e

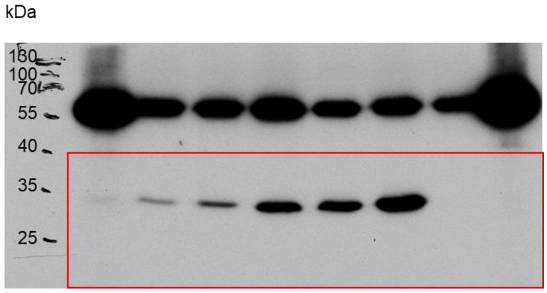

- Related to Figure 6c

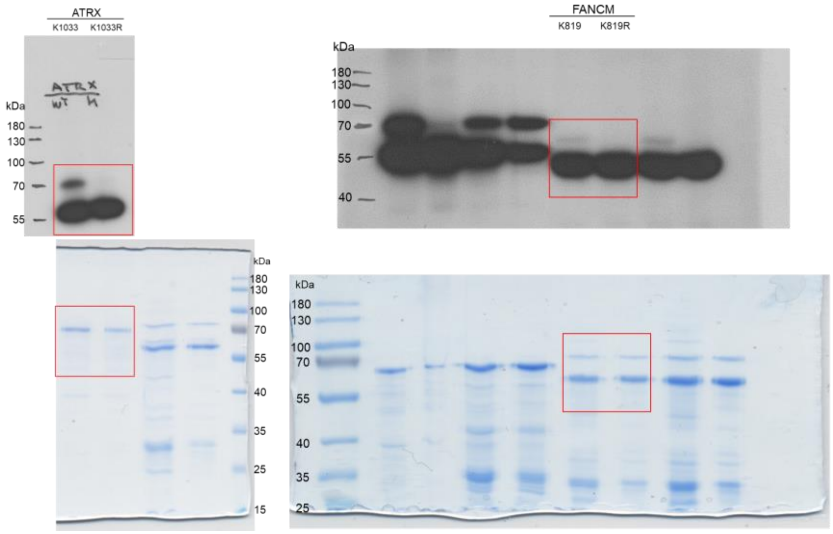

- Related to Figure 6d

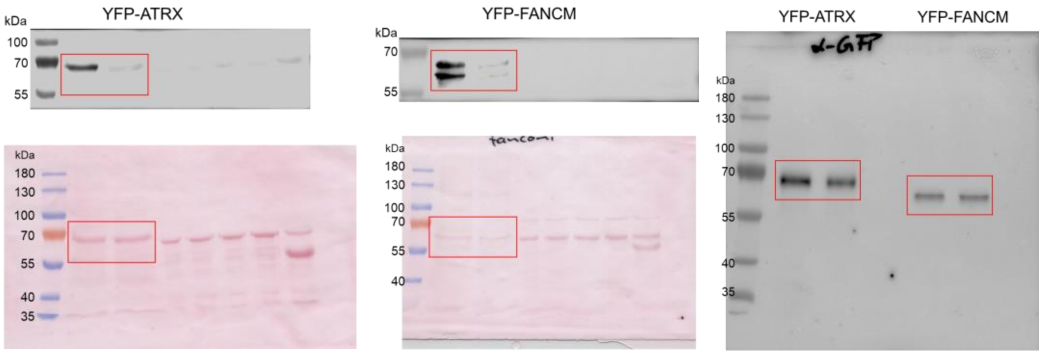

- Related to Supplementary Figure 1

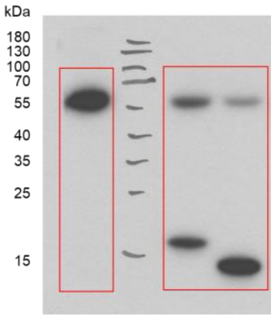

- Related to Supplementary Figure 5

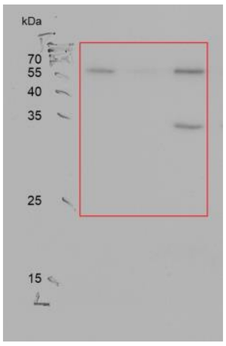

- Related to Supplementary Figure 8a

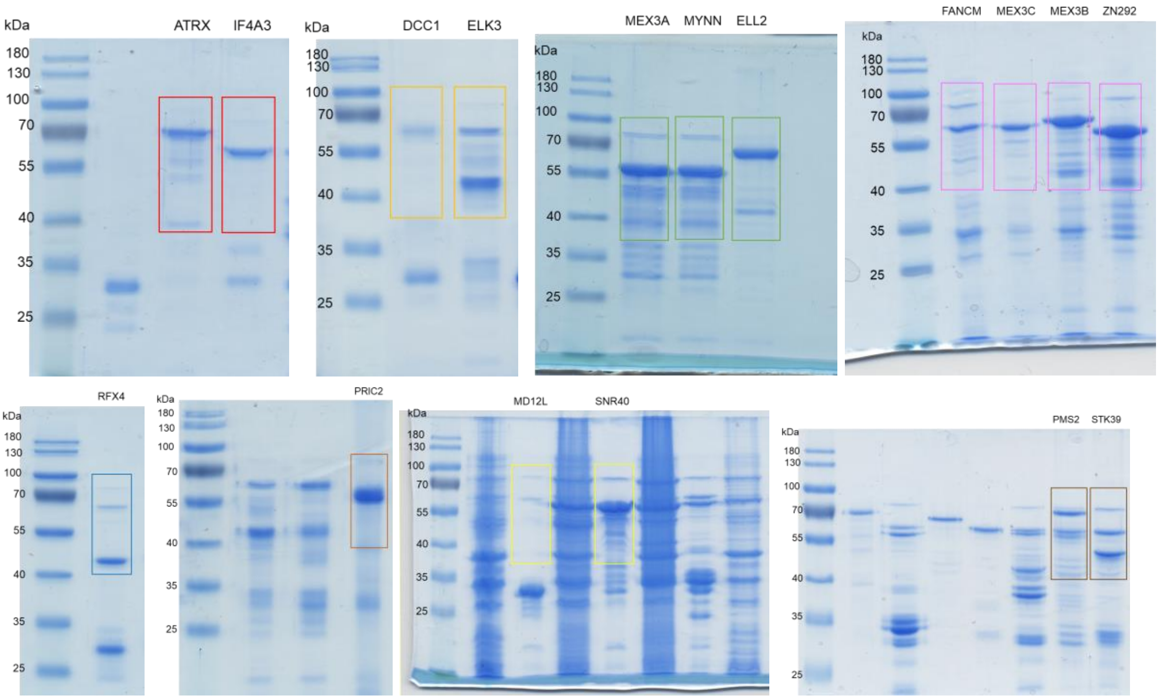

- Related to Supplementary Figure 8b

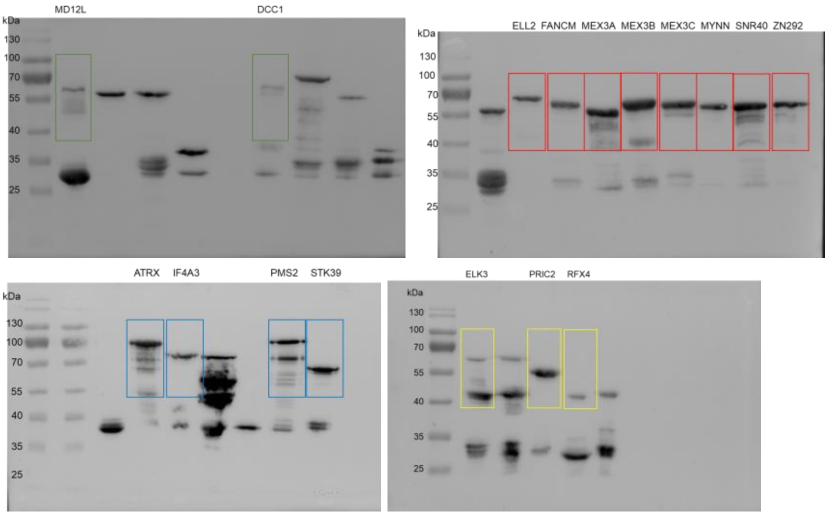

- Related to Supplementary Figure 8c

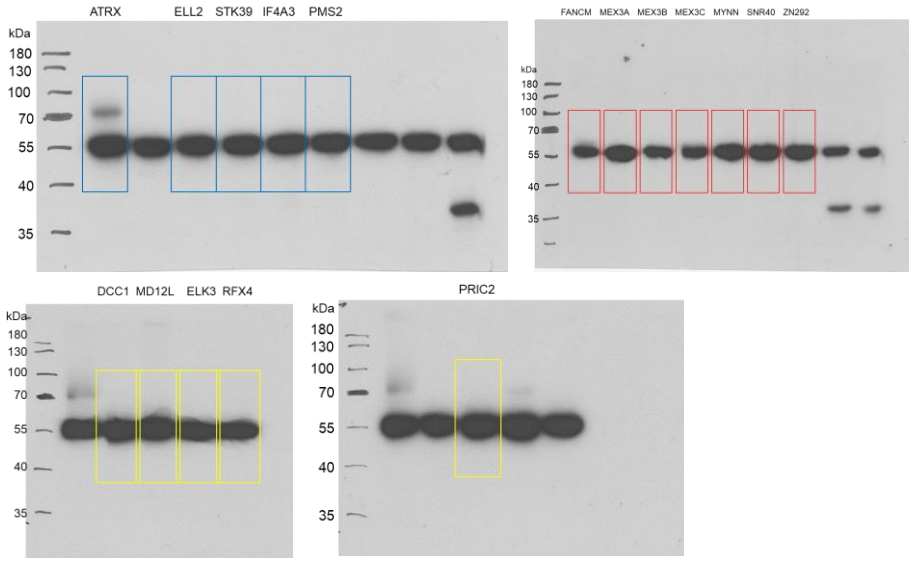

- Related to Supplementary Figure 9a

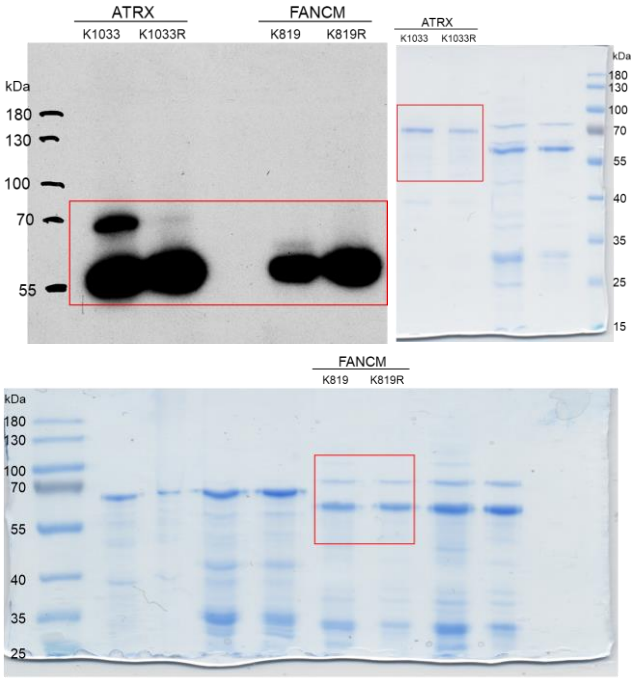

- Related to Supplementary Figure 9b

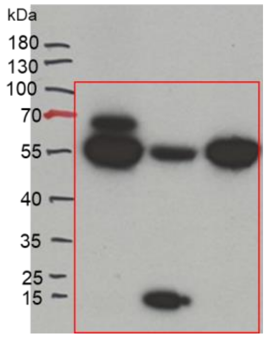

- Related to Supplementary Figure 10

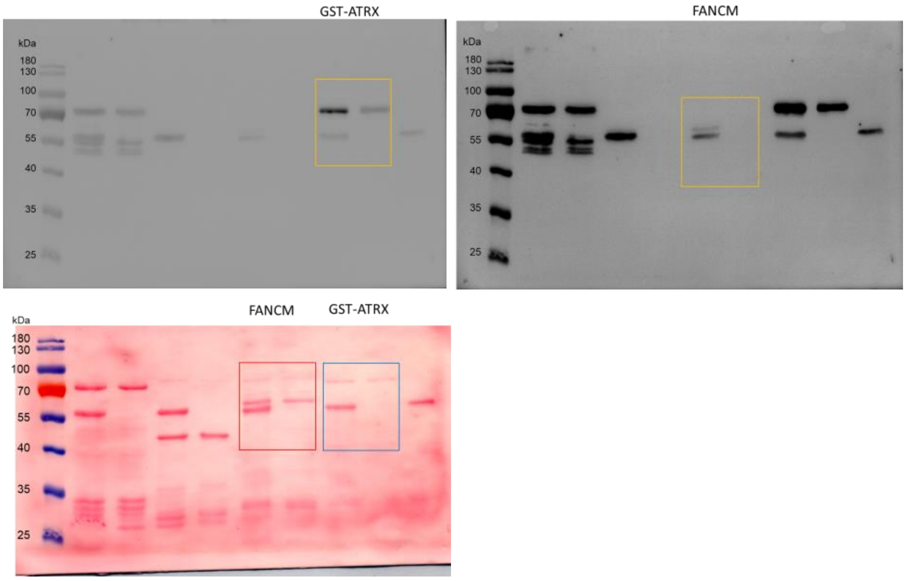

- Related to Supplementary Figure 11

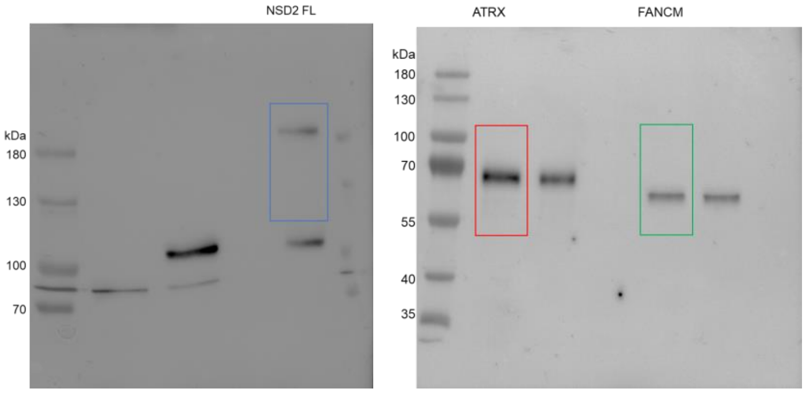

Supplementary Tables

Supplementary Table 1: Sequences of the peptide SPOT array shown in Figure 2a.

Spot position	ID	Sequence
A1	H3K36	A P A T G G V K K P H R Y R P
A2	H3K36A	A P A T G G V A K P H R Y R P
A3	H3A31G	A P G T G G V K K P H R Y R P
A4	H3A31H	A P H T G G V K K P H R Y R P
A5	H3A31K	A P K T G G V K K P H R Y R P
A6	H3A31M	A P M T G G V K K P H R Y R P
A7	H3T32G	A P A G G G V K K P H R Y R P
A8	H3T32K	A P A K G G V K K P H R Y R P
A9	H3T32F	A P A F G G V K K P H R Y R P
A10	H3T32V	A P A V G G V K K P H R Y R P
B1	H3G34F	A P A T G F V K K P H R Y R P
B2	H3V35I	A P A T G G I K K P H R Y R P
B3	H3V35L	A P A T G G L K K P H R Y R P
B4	H3K37R	A P A T G G V K R P H R Y R P
B5	H3K37Q	A P A T G G V K Q P H R Y R P
B6	H3K37I	A P A T G G V K I P H R Y R P
B7	H3K37V	A P A T G G V K V P H R Y R P
B8	H3P38I	A P A T G G V K K I H R Y R P
B9	H3P38L	A P A T G G V K K L H R Y R P
B10	H3P38V	A P A T G G V K K V H R Y R P
C1	H3H39N	A P A T G G V K K P N R Y R P
C2	H3H39G	A P A T G G V K K P G R Y R P
C3	H3H39L	A P A T G G V K K P L R Y R P
C4	H3H39S	A P A T G G V K K P S R Y R P
C5	H3R40N	A P A T G G V K K P H N Y R P
C6	H3R40H	A P A T G G V K K P H H Y R P
C7	H3R40L	A P A T G G V K K P H L Y R P
C8	H3R40K	A P A T G G V K K P H K Y R P
C9	H3K36	A P A T G G V K K P H R Y R P
C10	H3K36A	A P A T G G V A K P H R Y R P

Supplementary Table 2: Sequences of the peptide SPOT array shown in Figure 2b.

Spot position	ID	Sequence
A1	H3K36	A P A T G G V K K P H R Y R P
A2	H3K36A	A P A T G G V A K P H R Y R P
A3	H3K36_GS	A G A T G G V K K P H R Y R S
A4	H3K36A_GS	A G A T G G V A K P H R Y R S
A5		
A6	H3A31K_GS	A G K T G G V K K P H R Y R S
A7	H3K37R_GS	A G A T G G V K R P H R Y R S
A8	H3P38V_GS	A G A T G G V K K V H R Y R S
A9	H3H39N_GS	A G A T G G V K K P N R Y R S
A10	H3T32V_GS	A G A T G G V K K P H N Y R S
B1	two mutations GS	A G K T G G V K R P H R Y R S
B2		A G K T G G V K K V H R Y R S
B3		A G K T G G V K K P N R Y R S
B4		A G K T G G V K K P H N Y R S
B5		A G A T G G V K R V H R Y R S
B6		A G A T G G V K R P N R Y R S
B7		A G A T G G V K R P H N Y R S
B8		A G A T G G V K K V N R Y R S
B9		A G A T G G V K K V H N Y R S
B10		A G A T G G V K K P N N Y R S
C1	three mutations GS	A G K T G G V K R V H R Y R S
C2		A G K T G G V K R P N R Y R S
C3		A G K T G G V K R P H N Y R S
C4		A G K T G G V K K V N R Y R S
C5		A G K T G G V K K V H N Y R S
C6		A G K T G G V K K P N N Y R S
C7		A G A T G G V K R V N R Y R S
C8		A G A T G G V K R V H N Y R S
C9		A G A T G G V K R P N N Y R S
C10		A G A T G G V K K V N N Y R S
D1	four mutations GS	A G K T G G V K R V N R Y R S
D2		A G K T G G V K R V H N Y R S
D3		A G K T G G V K R P N N Y R S
D4		A G K T G G V K K V N N Y R S
D5		A G A T G G V K R V N N Y R S
D6		
D7	five mutations_GS	A G K T G G V K R V N N Y R S
D8	five mutations H3K36A_GS	A G K T G G V A R V N N Y R S
D9	five mutations	A P K T G G V K R V N N Y R P
D10	five mutations H3K36	A P K T G G V A R V N N Y R P

Supplementary Table 3: Sequences of the peptide SPOT array shown in Figure 2c.

Spot position	ID	Sequence
A1	H3K36	A P A T G G V K K P H R Y R P
A2	two mutations	A P A T G G V K K P N N Y R P
A3	three mutations	A P K T G G V K R P H N Y R P
A4		A P K T G G V K K P N N Y R P
A5		A P A T G G V K R P N N Y R P
A6		A P A T G G V K K V N N Y R P
A7	four mutations	A P K T G G V K R V H N Y R P
A8		A P K T G G V K R P N N Y R P
A9		A P K T G G V K K V N N Y R P
A10		A P A T G G V K R V N N Y R P
A11	five mutations	A P K T G G V K R V N N Y R P
B1	H3K36A	A P A T G G V A K P H R Y R P
B2	two mutations_K36A	A P A T G G V A K P N N Y R P
B3	three mutations_K36A	A P K T G G V A R P H N Y R P
B4		A P K T G G V A K P N N Y R P
B5		A P A T G G V A R P N N Y R P
B6		A P A T G G V A K V N N Y R P
B7	four mutations_K36A	A P K T G G V A R V H N Y R P
B8		A P K T G G V A R P N N Y R P
B9		A P K T G G V A K V N N Y R P
B10		A P A T G G V A R V N N Y R P
B11	five mutations_K36A	A P K T G G V A R V N N Y R P

Supplementary Table 4: Sequences of the peptide SPOT array shown in Figure 6b.

Swiss Prot No	ID	Protein name	Sequence	Target position
Q8IZT6	ASPM	Abnormal spindle-like microcephaly-associated protein	Q T Y F N K L K K I T K T V Q	2213
Q8IZT6	ASPM	Abnormal spindle-like microcephaly-associated protein	Q T Y F N K L A K I T K T V Q	2213
P46100	ATRX	Transcriptional regulator ATRX	C H F P K G I K Q I K N G T T	1033
P46100	ATRX	Transcriptional regulator ATRX	C H F P K G I A Q I K N G T T	1033
Q9UIF8	BAZ2B	Bromodomain adjacent to zinc fingerdomain protein 2B	M K Q Q E K I K R I Q Q I R M	948
Q9UIF8	BAZ2B	Bromodomain adjacent to zinc fingerdomain protein 2B	M K Q Q E K I A R I Q Q I R M	948
Q9BVC3	DCC1	Sister chromatid cohesion protein DCC1	R P K L K K L K K L L M E N P	139
Q9BVC3	DCC1	Sister chromatid cohesion protein DCC1	R P K L K K L A K L L M E N P	139
P09884	DPOLA	DNA polymerase alpha catalytic subunit	V E R R K Q V K Q L M K Q Q D	926
P09884	DPOLA	DNA polymerase alpha catalytic subunit	V E R R K Q V A Q L M K Q Q D	926
P41970	ELK3	ETS domain-containing protein Elk-3	Y Y D K N I I K K V I G Q K F	73
P41970	ELK3	ETS domain-containing protein Elk-3	Y Y D K N I I A K V I G Q K F	73
P28324	ELK4	ETS domain-containing protein Elk-4	Y Y V K N I I K K V N G Q K F	73
P28324	ELK4	ETS domain-containing protein Elk-4	Y Y V K N I I A K V N G Q K F	73
O00472	ELL2	RNA polymerase II elongation factor ELL2	H N K L A H I K R L I G E F D	625
O00472	ELL2	RNA polymerase II elongation factor ELL2	H N K L A H I A R L I G E F D	625
Q8IYD8	FANCM	Fanconi anemia group M protein	H K K S S F I K N I N Q G S S	819
Q8IYD8	FANCM	Fanconi anemia group M protein	H K K S S F I A N I N Q G S S	819
P38919	IF4A3	Eukaryotic initiation factor 4A-III	A I Q Q R A I K Q I I K G R D	70
P38919	IF4A3	Eukaryotic initiation factor 4A-III	A I Q Q R A I A Q I I K G R D	70

Q86YW9	MD12L	Mediator of RNA polymerase II transcription subunit 12-like protein	R A Y M N L V K K L K K E L G	1604
Q86YW9	MD12L	Mediator of RNA polymerase II transcription subunit 12-like protein	R A Y M N L V A K L K K E L G	1604
A1L020	MEX3A	RNA-binding protein MEX3A	G P K G A T I K R I Q Q Q T N	247
A1L020	MEX3A	RNA-binding protein MEX3A	G P K G A T I A R I Q Q Q T N	247
Q6ZN04	MEX3B	RNA-binding protein MEX3B	G P K G A T I K R I Q Q Q T H	184
Q6ZN04	MEX3B	RNA-binding protein MEX3B	G P K G A T I A R I Q Q Q T H	184
Q5U5Q3	MEX3C	RNA-binding protein MEX3C	G P K G A T I K R I Q Q Q T H	350
Q5U5Q3	MEX3C	RNA-binding protein MEX3C	G P K G A T I A R I Q Q Q T H	350
Q9NPC7	MYNN	Myoneurin	G N S Y T D I K N L K K H K T	512
Q9NPC7	MYNN	Myoneurin	G N S Y T D I A N L K K H K T	512
Q8N3S3	PHTF2	Putative homeodomain transcription factor 2	E V P H F R L K K V Q N I K M	574
Q8N3S3	PHTF2	Putative homeodomain transcription factor 2	E V P H F R L A K V Q N I K M	574
P54278	PMS2	Mismatch repair endonuclease PMS2	S S L A K R I K Q L H H E A Q	630
P54278	PMS2	Mismatch repair endonuclease PMS2	S S L A K R I A Q L H H E A Q	630
Q7Z3G6	PRIC2	Prickle-like protein 2	P G E K L R I K Q L L H Q L P	74
Q7Z3G6	PRIC2	Prickle-like protein 2	P G E K L R I A Q L L H Q L P	74
Q33E94	RFX4	Transcription factor RFX4	A K R Q G S L K K V A Q Q F L	421
Q33E94	RFX4	Transcription factor RFX4	A K R Q G S L A K V A Q Q F L	421
Q9NQR1	SETD8	Histone-lysine N-methyltransferase SET8	A I A K Q A L K K P I K G K Q	158
Q9NQR1	SETD8	Histone-lysine N-methyltransferase SET8	A I A K Q A L A K P I K G K Q	158
Q96DI7	SNR40	U5 small nuclear ribonucleoprotein 40 kDa protein	S E T G E R V K R L K G H T S	145
Q96DI7	SNR40	U5 small nuclear ribonucleoprotein 40 kDa protein	S E T G E R V A R L K G H T S	145
Q9H6I2	SOX17	Transcription factor SOX-17	R K Q V K R L K R V E G G F L	149
Q9H6I2	SOX17	Transcription factor SOX-17	R K Q V K R L A R V E G G F L	149

Q9UEW8	STK39	STE20/SPS1-related proline-alanine-rich protein kinase	R Q E R V A I K R I N L E K C	92
Q9UEW8	STK39	STE20/SPS1-related proline-alanine-rich protein kinase	R Q E R V A I A R I N L E K C	92
Q9Y3A2	UTP11	Probable U3 small nucleolar RNA-associated protein 11	V T N Q T G L K R I A K E R Q	189
Q9Y3A2	UTP11	Probable U3 small nucleolar RNA-associated protein 11	V T N Q T G L A R I A K E R Q	189
O60281	ZN292	Zinc finger protein 292	R Q K A S N L K R V N K E K N	2531
O60281	ZN292	Zinc finger protein 292	R Q K A S N L A R V N K E K N	2531

Supplementary Table 5: Compilation of the domain boundaries and properties of the cloned protein methylation substrate candidates. MW refers to the cloned GST fusion proteins as shown in Supplementary Figure 8. The validated substrates, ATRX and FANCM, are shaded in grey. Alphafold predictions were retrieved from the AlphaFold Protein Structure Database (https://alphafold.ebi.ac.uk/) [4].

Abbre-viation.	Swiss Prot No	Protein name	Cloned region	MW [kDa]	Target sequence	Target position	Structural context of target K
ATRX	P46100	Transcriptional regulator ATRX	893-1188	61.3	CHFPKGIK QIKNGTT	1033	Loop region (AlphaFold)
DCC1	Q9BVC3	Sister chromatid cohesion protein DCC1	13-278	57.9	RPKLKKLK KLLMENP	139	α-helix (AlphaFold)
ELK3	P41970	ETS domain-containing protein Elk-3	1-272	58.7	YYDKNIIK KVIGQKF	73	ß-sheet (AlphaFold)
ELL2	O00472	RNA polymerase II elongation factor ELL2	379-640	58.2	HNKLAHIK RLIGEFD	625	α-helix (pdb 5JW9)
FANCM	Q8IYD8	Fanconi anemia group M protein	723-933	51.9	HKKSSFIK NINQGSS	819	Loop region (AlphaFold)
IF4A3	P38919	Eukaryotic initiation factor 4A-III	2-280	59.2	AIQQRAIK QIIKGRD	70	α-helix (pdb 4C9B)
MD12L	Q86YW9	Mediator of RNA polymerase II transcription subunit 12-like protein	1486-1749	57.9	RAYMNLVK KLKKELG	1604	α-helix (AlphaFold)
MEX3A	A1L020	RNA-binding protein MEX3A	134-371	53.7	GPKGATIK RIQQQTN	247	α-helix (AlphaFold)
MEX3B	Q6ZN04	RNA-binding protein MEX3B	29-303	57.5	GPKGATIK RIQQQTH	184	α-helix (AlphaFold)
MEX3C	Q5U5Q3	RNA-binding protein MEX3C	224-478	55.6	GPKGATIK RIQQQTH	350	α-helix (pdb 5WWX)
MYNN	Q9NPC7	Myoneurin	372-603	53.6	GNSYTDIK NLKKHKT	512	α-helix (AlphaFold)
PMS2	P54278	Mismatch repair endonuclease PMS2	446-750	61.4	SSLAKRIK QLHHEAQ	630	α-helix (AlphaFold)
PRIC2	Q7Z3G6	Prickle-like protein 2	1-276	59.4	PGEKLRIK QLLHQLP	74	α-helix (AlphaFold)
RFX4	Q33E94	Transcription factor RFX4	300-556	46.3	AKRQGSLK KVAQQFL	421	α-helix (AlphaFold)
SNR40	Q96DI7	U5 small nuclear ribonucleo-protein 40 kDa protein	4-293	60.3	SETGERVK RLKGHTS	145	ß-sheet (PBD 5XJC)
STK39	Q9UEW8	STE20/SPS1-related proline-alanine-rich protein kinase	66-296	53.3	RQERVAIK RINLEKC	92	ß-sheet (AlphaFold)
ZN292	O60281	Zinc finger protein 292	2415-2655	55.1	RQKASNLK RVNKEKN	2531	No data available

Supplementary Table 6: Additional information about the MD simulated systems.

	**NSD2-H3K36-AdoMet**	**NSD2-ssK36-AdoMet**
number of simulations per system	50	50
simulation box dimensions	75 Å x 75 Å x 75 Å	75 Å x 75 Å x 75 Å
total number of atoms	61,128	62,503
total number of water molecules	10,000	10,000
salt concentration	0.1 M	0.1 M
lipid composition (number of molecules and type)	Not applicable	Not applicable

Supplementary references

1 Schuhmacher, M. K. *et al.* Sequence specificity analysis of the SETD2 protein lysine methyltransferase and discovery of a SETD2 super-substrate. *Communications biology* **3**, 511 (2020).

2 Schnee, P. *et al.* Mechanistic basis of the increased methylation activity of the SETD2 protein lysine methyltransferase towards a designed super-substrate peptide. *Communications Chemistry* **5**, 139 (2022).

3 Schnee, P., Pleiss, J. & Jeltsch, A. Approaching the catalytic mechanism of protein lysine methyltransferases by biochemical and simulation techniques. *Crit Rev Biochem Mol Biol*, 1-49 (2024).

4 Varadi, M. *et al.* AlphaFold Protein Structure Database: massively expanding the structural coverage of protein-sequence space with high-accuracy models. *Nucleic acids research* **50**, D439-D444 (2022).
