# Supplementary material for: Discovery of NSD2 non-histone substrates and design of a super-substrate
Source: Commun Biol. 2024 Jun 8;7:707. doi: 10.1038/s42003-024-06395-z (PMC11162472; doi:10.1038/s42003-024-06395-z)
Supplement: Supplementary file 3 — Description of Additional Supplementary Files [file 42003_2024_6395_MOESM3_ESM.pdf]

Description of Additional Supplementary Files

File name: Supplementary Data 1
Description: Sequences of the peptide SPOT array shown in Figure 6a.

File name: Supplementary Data 2
Description: The source data behind the graphs in the paper.
